# Combining Selective Pressures to Enhance the Durability of Disease Resistance Genes

**DOI:** 10.3389/fpls.2016.01916

**Published:** 2016-12-23

**Authors:** REX Consortium, Denis Bourguet

**Keywords:** durable disease plant resistance, strategy of resistance gene deployment, pyramids of plant resistance, gene stacking, *mosaic* of plant resistance, resistance breakdown, management of plant pathogens, fungicides

## Abstract

The efficacy of disease resistance genes in plants decreases over time because of the selection of virulent pathogen genotypes. A key goal of crop protection programs is to increase the durability of the resistance conferred by these genes. The spatial and temporal deployment of plant disease resistance genes is considered to be a major factor determining their durability. In the literature, four principal strategies combining resistance genes over time and space have been considered to delay the evolution of virulent pathogen genotypes. We reviewed this literature with the aim of determining which deployment strategy results in the greatest durability of resistance genes. Although theoretical and empirical studies comparing deployment strategies of more than one resistance gene are very scarce, they suggest that the overall durability of disease resistance genes can be increased by combining their presence in the same plant (pyramiding). Retrospective analyses of field monitoring data also suggest that the pyramiding of disease resistance genes within a plant is the most durable strategy. By extension, we suggest that the combination of disease resistance genes with other practices for pathogen control (pesticides, farming practices) may be a relevant management strategy to slow down the evolution of virulent pathogen genotypes.

Go back young man and gather up your weary and defeated genes of the past, take your currently successful genes, find some new ones if you can, and build yourself a genetic pyramid.

(Nelson, 1978, p. 376)

## The Need for Crop Protection

Pathogens and pests, from viruses to insects and weeds, threaten crops (Strange and Scott, 2005; Fisher et al., 2012; Oerke et al., 2012), human health (Wolfe et al., 2007; Dunn et al., 2010), and ecosystems (Crowl et al., 2008) to such a point that we go to considerable efforts to control them. This control is likely to become increasingly important in the future, because it has been predicted that human population growth and changes in dietary habits will lead to a doubling of food demand in the next few decades (Chakraborty and Newton, 2011; Tilman et al., 2011). However, this control is also likely to become more difficult to achieve, because potential crop losses due to pests and diseases are expected to increase with global changes, including climate change (Chakraborty and Newton, 2011), human-mediated invasions and disease re-emergence (Anderson et al., 2004). Pathogens currently cause losses of 10–16% of the global harvest. Decreasing this percentage is a priority for the achievement of food security (Chakraborty and Newton, 2011).

Crop pathogens can be controlled by several methods, including the cultivation of plants bearing resistance genes (Hammond-Kosack and Jones, 1997), the use of pesticides (Smith and Pimentel, 1978; Matthews et al., 2014), the conservation or management of biological control (Tjamos et al., 2013) and the implementation of prophylactic measures (Gomez et al., 2007). Unfortunately, pathogen populations can develop resistance in response to all these control methods (McDonald and Linde, 2002a; REX Consortium, 2007; Gray et al., 2009; Lannou, 2012; Bardin et al., 2015).

## Pathogen Evolution in Response to Disease Resistance Genes

Historically, two categories of disease resistance have been recognized in plants: qualitative and quantitative resistance. Qualitative resistance is genetically controlled by major genes, which provide phenotypically complete or incomplete resistance to the pathogen. It is based on gene-for-gene interactions, in which the protein encoded by the avirulence gene of the pathogen is specifically ‘recognized’ by the product of the corresponding resistance gene of the plant (Flor, 1971). This recognition is followed by a hypersensitive response in the plant, restricting the pathogen to the primary infection site. The deployment of major genes conferring qualitative resistance is generally followed by the evolution of matching virulence in the pathogen, in so-called ‘boom-and-bust’ cycles (Johnson, 1961; Parlevliet, 1989). Quantitative resistance is generally controlled by multiple genetic factors (minor genes) in the plant providing partial resistance to the pathogen and leading to a decrease in symptom severity and/or the progress of epidemics over time (Poland et al., 2009; St. Clair, 2010). There is also growing evidence for the selection of pathogen genotypes able to overcome quantitative resistance (Burdon et al., 2014).

## Durability of Disease Resistance Genes

Disease resistance genes are a limited resource and their introduction into new varieties is costly. The preservation of their efficacy over time (i.e., their durability) is, thus, a tremendous challenge. Johnson (1981) defined durable disease resistance in plants as a resistance that remain effective while a cultivar possessing it is widely cultivated. The durability of a disease resistance gene can be measured by the time required for the selection of pathogen genotypes overcoming the resistance and thereby rendering the resistance gene ineffective. The pathogen genotype frequency that must be reached for the resistance to be considered broken down is clearly arbitrary and depends on the socio-economic context. Durability is dependent on intrinsic properties of the pathosystem, such as the mechanism and genetics of the molecular interaction and the biology of the targeted pathogen (e.g., ploidy, reproduction mode, mutation rate) (Brown, 2015). However, it also depends on external factors (environmental conditions and agronomic practices) affecting the fitness of the organisms targeted (Brown, 2015). Once the resistance genes have been introduced into plant varieties, their efficacy can be preserved only by manipulating these external factors.

## Strategies for the Deployment of Disease Resistance Genes

The deployment of disease resistance genes in the field is a major external factor affecting their durability. The availability of more than one source of resistance genes at a given time provides opportunities for strategies based on the pattern of deployment of these genes over space and time. Such strategies can be classified into four categories (**Table 1**): (1) the incorporation of several resistance genes into the same plant (*pyramiding*), (2) the use of several different resistance genes in different plants within (*multiline* and *variety mixtures*) or between (*regional or landscape deployment*) fields, (3) the periodic alternation of different resistance genes at the same site (*rotation*) and (4) the use of each resistance gene until the breakdown of the resistance conferred and its replacement with a new resistance gene (*sequential release*).

**Table 1:** Description of the different strategies for disease resistance gene deployment over space and time, with the names used to define them and the names of the analogous strategies used to manage the evolution of resistance to both antibiotics in human and animal diseases and pesticides in agricultural pests and pathogens.

Description of the strategy	Spatial variation of resistance genes	Temporal variation of resistance genes	Names of the strategy and references^∗^	Names of the analogous strategies for antibiotics and pesticides^∗∗^
Combination of two or more resistance genes in one plant	No	No	***Pyramiding*** ^(1)^, Multigene varieties ^(1)^, Stacking ^(2)^	***Combination***
Mixture of several lines bearing different resistance genes within one field	Yes	No	***Multilines*** ^(3)^, Multiline cultivars ^(4)^, Composite varieties ^(5)^	***Mosaic***
Mixture of several cultivars with different resistance genes within one field	Yes	No	Cultivar mixtures ^(6)^, Multi blend varieties ^(7)^, Mass reservoirs ^(8)^, ***Variety mixtures*** ^(9)^	***Mosaic***
Plants bearing different resistance genes grown at the same time in different fields (at farm level or at landscape scale)	Yes	No	Regional or ***landscape deployment*** ^(10)^	***Mosaic***
Periodic use of different resistance genes	No	Yes	***Rotation*** ^(10)^, Alternation ^(11)^	***Periodic application***
Sequential use of different resistance genes but without a cycle. In this approach, a gene is used continuously until the evolution of virulence, after which a new gene is introduced, and so on.	No	Yes	***Sequential release*** ^(7)^, Plug-Plant-Pray ^(10)^	***Responsive alternation***


*The strategy names used in the text are reported in bold. ^∗^^(1)^ (Watson and Singh, 1952), ^(2)^ (Halpin, 2005), ^(3)^ (Jensen, 1952; Jensen, 1965), ^(4)^ (Borlaug, 1958; Browning and Frey, 1969), ^(5)^ (Borlaug, 1953), ^(6)^ (Wolfe and Barrett, 1980), ^(7)^ (Adugna, 2004), ^(8)^ (Allard and Hansche, 1964), ^(9)^ (Wolfe, 1985), ^(10)^ (McDonald and Linde, 2002b), ^(11)^ (Djian-Caporalino et al., 2014). ^∗∗^ The names of the strategies are those proposed by the REX Consortium (2013).*

## Toward A Theoretical Ranking of the Strategies Combining Disease Resistance Genes

From an evolutionary point of view, for the targeted organisms, genes conferring plant resistance are not conceptually different from pesticides. First, pathogens and pests are confronted with the same basic evolutionary forces (mutation, selection, migration, genetic drift, and recombination). Second, plant disease resistance genes and pesticides may both reduce the fitness of the targeted organisms. They exert a selection pressure on populations, triggering the evolution of virulence in the case of disease resistance genes (Sacristan and Garcia-Arenal, 2008; Brown, 2015) and the evolution of resistance in the case of pesticides (REX Consortium, 2013). Transgenic crops producing *Bacillus thuringiensis* (*Bt*) toxins, which are now widely cultivated (James, 2007), or RNAi carefully chosen to silence crucial genes in target pests, which are currently being developed and tested in field trials (Debat and Ducasse, 2014), provide a perfect illustration of the similarity between pesticides and plant disease resistance genes. They bridge the gap between these two control methods as they can be seen both as plants bearing disease resistance genes and as plants directly synthesizing pesticides in their tissues. As already reported for pesticides and disease resistance genes, resistance to *B. thuringiensis* crops has been found in many targeted pest populations (Tabashnik et al., 2013).

For pesticide management, four different strategies (*combination*, *mosaic*, *periodic application*, and *responsive alternation*, see **Table 1**) for increasing the durability of the molecules have already been compared theoretically and, to a lesser extent, empirically (REX Consortium, 2013). Provided that molecules have non-redundant modes of action, that they are used at full dose and that resistance has not yet evolved, these studies suggest the following hierarchy of strategies, in terms of the durability of the molecules: *combination* > *mosaic* = *periodic application* > *responsive alternation* (REX Consortium, 2013). This hierarchy has been explained by the probability of a given pest and its offspring being exposed to several pesticides. The durability of a given pesticide is expected to increase monotonously with this probability, which depends on the strategy used and has been referred to as the ‘degree of treatment heterogeneity’ (*DTH*) (see Suppelementary Material; REX Consortium, 2013).

The four strategies used in pesticide management fully mirror those used for plant resistance management (**Table 1**). Consequently, based on the results of the REX Consortium (2013), we hypothesize that the strategies can be ranked as follows, in terms of disease resistance gene durability: *pyramiding* > (*multilines, variety mixtures, and landscape deployment*) = *rotation* > *sequential release*. We would therefore expect pyramiding to be the most durable strategy given the low probability of virulence emerging with this system. This probability is low because it is the product of the probabilities of breaking down simultaneously each of the resistance genes.

## Comparisons of Strategies for the Short-Term Control of Pathogens Rather Than Durability

Many studies have compared strategies of disease resistance gene deployment. However, most focused on pathogen control over short periods of time (one or a few crop seasons or a limited number of pathogen generations) and did not take into account that the pathogen population might evolve (e.g., Brown et al., 1996; Porter et al., 2000; Maqbool et al., 2001; Jahier et al., 2009; Tan et al., 2010; Brunner et al., 2012; Obala et al., 2012; Zeller et al., 2012; Cingel et al., 2014; McCarville et al., 2014; Rule et al., 2014; Vu et al., 2014; Fukuoka et al., 2015). These comparative studies did not actually compare the durability of resistance genes. Along the same lines, Burdon et al. (2014) pointed that “*what is lacking are careful assessments (both empirical and theoretical), using ecological and evolutionary principles, of the most effective disease resistance deployment strategies (including spatial considerations) that will maximize both the short-term epidemiological and the longer-term evolutionary benefits of different combination strategies.*” However, assessing the durability of a given plant–pathogen interaction ultimately requires long-term experiments performed at the regional scale or over an even larger scale, which is notoriously difficult.

## Comparison of Strategies for Combining Disease Resistance Genes

There are many studies that compared the benefits of pyramiding resistance genes with those of the deployment of a single resistance gene; they found that the pyramiding strategy was more durable than the use of a single resistance gene (e.g., Vu et al., 2014). In particular, it has been shown that the addition of quantitative resistances can greatly increases the durability of major resistance genes (Palloix et al., 2009; Brun et al., 2010; Delourme et al., 2014). However, as pointed out by Mundt (2014), “*given constant crop area, it is logical that a resistance gene will last longer in mixture than in pure stand simply owing to reduced exposure to the pathogen (…). A more relevant question may be whether a given number of genes will last longer in mixtures than by sequential use in pure stand.*” Actually, there are very few studies that have addressed this issue. Sapoukhina et al. (2009) modeled scenario of spatial deployment of resistance genes and found that the evolutionary dynamics of the pathogen was not different for the pyramid of two resistance genes than for the random mixture of single-gene resistant plant genotypes. Djian-Caporalino et al. (2014) compared experimentally three strategies of disease resistance gene deployment — pyramiding, variety mixtures, and rotation — over a 3-year period. They found that the genes conferring resistance to root-knot nematodes in pepper and lettuce were more durable if deployed in a pyramiding system than if used in cultivar mixtures and rotations.

In addition to these comparisons of strategies, retrospective analyses have suggested that *DTH* maximization, *via* pyramiding, increases the durability of disease resistance genes. For example, in wheat, the deployment of single major genes conferring resistance to yellow rust was found to have been effective for relatively short periods of time in most places (Bayles et al., 2000). However, several wheat cultivars grown in Western Europe remained resistant for more than 15 years, and some of these varieties are still resistant to this rust. This was attributed to the pyramiding of several resistance genes in these cultivars. Subsequent genetic analysis of these cultivars revealed that they indeed contained combinations of resistance genes/QTL expressed in both seedlings and adult plants (de Vallavieille-Pope et al., 1990; Chen and Wang, 1996; Mallard et al., 2005; Jagger et al., 2011; Agenbag et al., 2012; Powell et al., 2013; Basnet et al., 2014). Similarly, in winter wheat it is believed that the durable resistance to stem rust and powdery mildew have been achieved by the combination of multiple minor resistance genes (Singh et al., 2011; Ellis et al., 2014; Brown, 2015).

Extensive practical experience has clearly demonstrated that, on average, genetically quantitative resistance is more durable (Parlevliet, 1989) probably because quantitative resistance is polygenic, involving many genes, whereas qualitative resistance is dependent on a single major resistance gene (Mundt, 2014). As Mundt (2014) explained, “*the accumulated wisdom of plant breeders has often been underestimated. Genes that contribute to durable pyramids /…/ were uncovered through the experience of breeders working in the field, and many more such combinations are sure to be found.*” It is equally plausible that quantitative resistance is more durable because it decreases selection intensity on the pathogen compared to major gene bringing total resistance.

## Pyramiding Disease Resistance Genes to Enhance Their Durability and Related Issues

Taken together, these theoretical considerations, empirical results and retrospective analyses converge in that pyramiding is likely the most powerful approach to provide durable resistance to plant pathogens. However, the efficacy of pyramiding could be compromise if several key assumptions are not met: (i) mutations to virulences are independent, (ii) virulences do not pre-exist in the pathogen population, (iii) resistance conferred by each pyramided genes have not been broken down before their deployement, (iv) combines different sources of genetic resistance with non-redundant modes of action. Furthermore, the advantage of pyramiding may also be reduced when the pathogen undergoes efficient sexual reproduction so that virulence genes can be resorted by recombination (Mundt, 1991; Burdon et al., 2014; Mundt, 2014; Brown, 2015).

Until recently, one of the major restrictions to pyramiding for seed companies and breeders was the time required to obtain a successful marketed variety. However, new approaches, including marker-assisted selection, genetic transformation, new sequencing technologies and genomic editing, have opened up new possibilities for breeders (Gupta et al., 2010; Lusser et al., 2012; Michelmore et al., 2013; Wang et al., 2014). They have promoted the discovery of new resistance genes and strongly facilitate their combination in single variety, by genomic selection (e.g., Heffner et al., 2009; Tester and Langridge, 2010). In the future, biotechnologies will allow creating genetically modified new variety with resistance alleles to which the pathogen has never been exposed (McDonald, 2014; Wulff and Moscou, 2014; Figueroa et al., 2016). We could therefore imagine that the deployment of resistance genes into new cultivars will be informed by the knowledge of population genetics of the corresponding avirulence genes and by the dynamics of these virulences in field pathogen populations (Wulff and Moscou, 2014; Figueroa et al., 2016).

However, breeders are still faced with several other difficulties (Burdon et al., 2014), including genes masking the expression of resistance conferred by another gene, genotype x environment interactions affecting resistance expression and the resulting phenotypes, and physiological costs associated with resistance genes that might impair agronomic performance.

It should be borne in mind that increasing the durability of disease resistance genes might not necessarily minimize the risk of resistance breakdown, which is calculated as the product of the probability of breakdown and its economic impact. The probability of breakdown is the probability of a “multi-virulent” strain, also referred as “super race,” of the pathogen emerging. The economic impact is the financial cost due to disease development following the breakdown of resistance plus the cost of developing a new effective strategy for keeping the disease under control. Maximizing *DTH* minimizes the probability of breakdown, but it may also increase the economic impact of resistance breakdown, which would be associated with the simultaneous loss of several disease resistance genes. The economic impact would be even greater if no other disease resistance genes were available in commercial varieties for use at a time of multi-virulent strain emergence.

## Combining Disease Resistance Genes with Other Means of Disease Control to Increase Their Durability

The durability of disease resistance genes could also be enhanced by combining them with other means of disease control, because the simultaneous use of several ‘weapons’ maximizes *DTH* (see Supplementary Material). For instance, the durability of a resistance gene targeting a pathogenic fungus may be increased by applying fungicides targeting the same pathogen. Unfortunately, very few models or empirical studies have considered such combinations of heterogeneous selective pressures to delay virulence emergence. Iacono et al. (2013) showed, with a theoretical approach, that the durability of a resistant cultivar is increased by superimposing a source of demographic stochasticity, such as intermittent applications of a fungicide. Similarly, Onstad et al. (2013) developed a theoretical model integrating pesticides, parasitoids, and transgenic insecticidal crops to control the diamond back moth, *Plutella xylostella*. They showed that the various means of pest control synergistically increased each other’s durability. There is a crucial need for additional studies to explore the power and limitations of such combinations in more detail. One of the limitations could be the use of pesticides. Indeed, despite their benefits in term of plant protection, their impact on human health and the environment (Bourguet and Guillemaud, 2016) might be, in some cases, too high to be acceptable by stakeholders.

**FIGURE 1:**
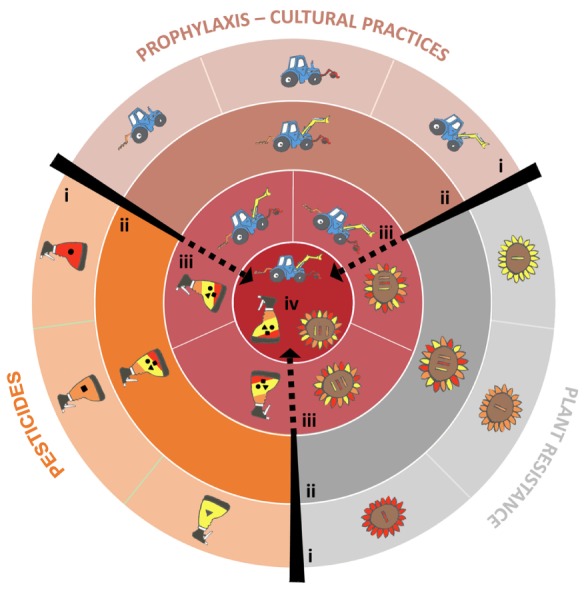
**How to combine the various methods available to control plant pathogens to maximize durability.** The three main categories of methods are presented by different symbols: a plant for plant resistance genes, a sprayer for pesticides, a tractor for farming methods. Strategies are obtained by combining these methods of pathogen management. The figure is constructed as a target, whose center maximizes the ‘Degree of treatment heterogeneity’ (*DTH*, see Supplementary material 1). (i) Only one method (either a plant resistance gene, an antifungal mode of action or a prophylaxis method) is used to control a plant pathogen species. (ii) Several methods of the same type (several plant resistance genes, or several antifungal modes of actions, or several prophylaxis methods) are combined. (iii) Two methods of different types are combined. (iv) All possible methods are combined. This strategy maximizes DTH, and maximizes the durability of plant resistance genes and antifungals.

More generally, agricultural practices designed to control a given pathogen (such as prophylactic methods, disease resistance genes, pesticides, biological control or the use of beneficial organisms) should, theoretically, be combined to increase their respective and overall durability (Ratnadass et al., 2012, **Figure 1**). In practice, such combinations are constrained by financial, organizational, human health and environmental factors that should be assessed on a case-by-case basis (Vanloqueren and Baret, 2008). Finally, it would be a good time to update the advice provided by Nelson in 1978 as follows: *Go back once more young man and gather up not only your most efficient genes, but also molecules, natural enemies and practices, and build a highly durable strategy … and be wise enough to make this strategy economically and ecologically sustainable*.

## Author Contributions

DB, FD, PF, TG, XR, CV, A-SW designed this research and equally participate in analyzing the data and writing the article.

## Conflict of Interest Statement

The authors declare that the research was conducted in the absence of any commercial or financial relationships that could be construed as a potential conflict of interest.
