# Supplementary material for: Combining Selective Pressures to Enhance the Durability of Disease Resistance Genes
Source: Front Plant Sci. 2016 Dec 23;7:1916. doi: 10.3389/fpls.2016.01916 (PMC5180194; doi:10.3389/fpls.2016.01916)
Supplement: Supplementary file 1 [file Presentation_1.PDF]

Supplementary Material

Degree of treatment heterogeneity (*DTH*)

Let us consider that K disease resistance genes ($K \geq 2$) targeting a pathogen population are deployed. Let us assume that N virulent strains ($1 \leq N \leq K$), each able to circumvent one resistance gene, are present in the pathogen population at the time of deployment. According to the REX Consortium (2013), *DTH* can be defined as the probability that these virulent strains encounter more than one resistance gene over the course of two generations and disappear because of them:

$$DTH = \prod_{i=1}^{N} (1 - S_{1i} S_{2i})$$

where S_{1i} and S_{2i} are the survival probabilities of the virulent strain adapted to disease resistance gene i during the first and second generation, respectively.

For the sake of simplicity, let us assume that the pathogen species is haploid, and that no mutation or recombination occurs over the two generations. The duration of each generation is t_G. The virulent strain i may be anywhere in the environment at the beginning of the first generation. It grows on its host plant and then reproduces after a period t_G - 1, giving rise to reproductive structures capable of dispersal. It has a survival probability at each time t of 1 when faced with disease resistance gene i and of (1 - s) for all other disease resistance genes, s ($0 < s \leq 1$) being the intensity of the selection exerted by each disease resistance gene.

In the *Pyramiding* strategy, all K disease resistance genes are present in each plant, everywhere and at all times t. We thus have:

$$S_{1i} = S_{2i} = (1-s)^{t_G(K-1)} \text{ for all } i$$

$$\text{and } DTH_{Pyr} = (1-(1-s)^{2t_G(K-1)})^N$$

In the *Rotation* strategy, the *K* disease resistance genes are used successively, over the whole environment, with a period of rotation *T*, and T/K is the duration of use of each disease resistance gene within a given period. The generation time of the pathogen t_G is usually much shorter than the duration of the of each disease resistance gene ($T/K \gg t_G$). Therefore, two resistance genes at most may be used during this time ($T/K > 2t_G$). The probability of a new resistance gene being used over the course of two generations of the pathogen is $1/(\left\lfloor \frac{T}{K}/2t_G \right\rfloor + 1)$ (with $\lfloor x \rfloor$ the largest integer not greater than *x*). Let us assume that the change from resistance gene i to j occurs between the two generations of the pathogen. In this case,

$$S_{1i} = 1 \text{ and } S_{2i} = (1-s)^{t_G}, \text{ so } 1 - S_{1i}S_{2i} = 1 - (1-s)^{t_G}$$

$$S_{1j} = (1-s)^{t_G} \text{ and } S_{2j} = 1, \text{ so } 1 - S_{1j}S_{2j} = 1 - (1-s)^{t_G}$$

$S_{1k} = S_{2k} = (1-s)^{t_G}$, so $1 - S_{1k}S_{2k} = 1 - (1-s)^{2t_G}$ for all $k \neq$ □ or j

$$\text{Thus, } DTH_{rot} = \frac{1}{\left(\left\lfloor \frac{T}{K}/2t_G \right\rfloor + 1\right)} (1 - (1-s)^{t_G})^2 (1 - (1-s)^{2t_G})^{N-2}$$

In the *Spatial deployment* strategy, the *K* disease resistance genes are used continuously, in different patches. The relative area protected by each resistance gene is $1/K$.

$$S_{1i} = \frac{1}{K} + \left(1 - \frac{1}{K}\right)(1-s)^{t_G} \text{ and } S_{2i} = X_i + (1 - X_i)(1-s)^{t_G} \text{ for all } i$$

with X_i the probability that the reproductive structures disperse on a plant expressing disease resistance gene *i*. In the case of uniform dispersal, $X_i = 1/K$ for all *i*.

$$DTH_{spat} = \left(1 - \left(\frac{1}{K} + \left(1 - \frac{1}{K}\right)(1-s)^{t_G}\right)^2\right)^N$$

Under these assumptions, the *Pyramiding* strategy yields the highest *DTH*, followed by *Spatial deployment* and *Rotation*.

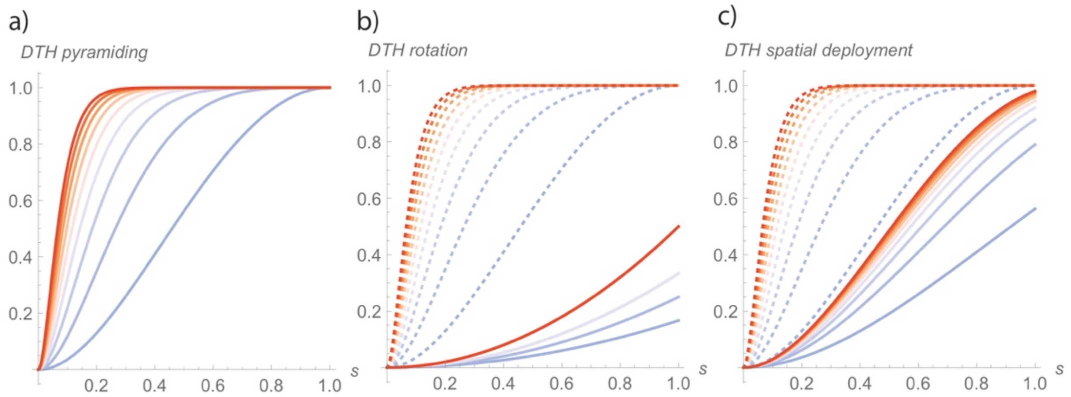

Legend of the figure: 'Degree of treatment heterogeneity' (*DTH*) experienced by two resistant genotypes arising through mutation in a pathogen population, as a function of the number of selective pressures (from 2 to 10, represented by solid lines in shades of blue to red) and the intensity (*s*) of each selective pressure. *DTH* is calculated for a) the *Pyramiding* strategy b), the *Rotation* strategy, and c) the *Spatial deployment* strategy, assuming uniform dispersal. In b) and c), *DTH* for the *Pyramiding* strategy is indicated by dashed lines for comparison. In b) the period of rotation is 20 times the generation time of the pathogen. Selective pressures may be disease resistance genes and/or other means of pathogen control (pesticides, farming practices).

The superiority of *Pyramiding* is accounted for by the higher intensity of selection exerted by each plant on pathogen strains, due to the simultaneous expression of several resistance genes. At each time step, the local intensity of selection is *Ks* for the *Pyramiding* strategy, versus *s* for the other two strategies. In other words, with the *Pyramiding* strategy, the pathogen strain may be killed simultaneously by *K* selective pressures ('multiple intragenerational killing'; REX Consortium, 2013). The relative ranks of *Rotation* and *Spatial deployment* may vary in practice, depending on the number of disease resistance genes that can actually be deployed over two generations of the pathogen. In the case of the *Rotation* strategy, *K* is usually close to 1 for most plant-pathogen systems, because the pathogen has a much shorter generation time than the plant. In this case, DTH_{rot} is close to 0. However, if very short rotations of the various disease resistance genes were possible (e.g., by using plants containing the various resistance genes, with alternating gene expression mechanisms), then the *K* disease resistance genes could be used successively over the whole environment during the two pathogen generations. In this improbable case, the duration of use of each resistance gene would be $2t_G/K$ and $DTH_{rot} = (1-(1-s)^{2t_G\left(1-\frac{1}{K}\right)})^N$, and *Rotation* might then outperform *Spatial deployment* for some parameters.
